# Quantitative modeling of carcinogenesis induced by single beams or mixtures of space radiations using targeted and non-targeted effects

**DOI:** 10.1038/s41598-021-02883-y

**Published:** 2021-12-06

**Authors:** Igor Shuryak, Rainer K. Sachs, David J. Brenner

**Affiliations:** 1 Center for Radiological Research, Columbia University Irving Medical Center, 630 West 168th St., New York, NY 10032 USA; 2 Department of Mathematics, University of California, Berkeley, CA 94720 USA

**Keywords:** Biophysics, Cancer, Computational biology and bioinformatics

## Abstract

Ionizing radiations encountered by astronauts on deep space missions produce biological damage by two main mechanisms: (1) Targeted effects (TE) due to direct traversals of cells by ionizing tracks. (2) Non-targeted effects (NTE) caused by release of signals from directly hit cells. The combination of these mechanisms generates non-linear dose response shapes, which need to be modeled quantitatively to predict health risks from space exploration. Here we used a TE + NTE model to analyze data on APC^(1638N/+)^ mouse tumorigenesis induced by space-relevant doses of protons, ^4^He, ^12^C, ^16^O, ^28^Si or ^56^Fe ions, or γ rays. A customized weighted Negative Binomial distribution was used to describe the radiation type- and dose-dependent data variability. This approach allowed detailed quantification of dose–response shapes, NTE- and TE-related model parameters, and radiation quality metrics (relative biological effectiveness, RBE, and radiation effects ratio, RER, relative to γ rays) for each radiation type. Based on the modeled responses for each radiation type, we predicted the tumor yield for a Mars-mission-relevant mixture of these radiations, using the recently-developed incremental effect additivity (IEA) synergy theory. The proposed modeling approach can enhance current knowledge about quantification of space radiation quality effects, dose response shapes, and ultimately the health risks for astronauts.

## Introduction

Deleterious health effects from a multi-component mixture of sparsely ionizing and densely ionizing radiations^1–3^ represent an important challenge which needs to be addressed to enable long-duration space exploration missions such as a trip to Mars. Densely ionizing heavy ions are particularly important in this context because they produce spatially clustered ionizations along defined tracks^4^, whereas sparsely ionizing γ rays generate more random distributions of ionizations. Such energy deposition differences between radiation types influence their biological effectiveness. Specifically, damage induced by heavy ions tends to result in more frequent and severe adverse health effects including carcinogenesis, relative to sparsely ionizing radiations^5–7^.

Ionizing radiations cause biological damage in multiple ways, which can be usefully classified into two categories: (1) Targeted effects (TE), involving the consequences of direct traversals of cells by ionizing tracks leading to DNA double strand breaks and other lesions. (2) Non-targeted effects (NTE, also called “bystander” effects) caused by release of signals from cells directly hit by the tracks and the effects of these signals on other cells.

In deep space, low linear energy transfer (LET) protons are much more abundant than high-LET heavy ions^1^. A given cell nucleus is unlikely to be traversed by a heavy ion track core more than once during a space mission such as a trip to Mars and back, and most cell nuclei will not be traversed at all. In such stochastic exposure scenarios, where the probability of TE induced by heavy ion track cores is relatively low, NTE are likely to be the dominant mechanism producing adverse health effects associated with heavy ions^1^. The NTE term includes multiple types of effects^6,8–11^. For these reasons, there is a diverse and growing NTE-related literature, including quantitative mathematical models, e.g.^12–14^. A detailed review of NTE models is beyond the scope of this study, and we attempted it elsewhere^11^. As a simplified generalization for the purposes of mathematical modeling, here we assume that NTE responses can induce susceptible cells to switch from a normal state to an “activated” state, e.g*.* of persistent oxidative stress^15–18^.

The combination of TE and NTE in the radiation response can cause dose response shapes for space radiations to become markedly non-linear, particularly in the dose range relevant for space missions (i.e*.* several cGy of heavy ions). Mechanistically-motivated radiation carcinogenesis models serve as important tools to describe these dose response shapes and magnitudes, and to quantitatively predict cancer risks from space exploration. Here we used a modification of our model that includes both TE and NTE^19^ to analyze a large data set on APC^(1638N/+)^ mouse tumorigenesis induced by space-relevant doses of protons, ^4^He, ^12^C, ^16^O, ^28^Si or ^56^Fe ions, or γ rays. This data set became much larger and more detailed since our previous analysis^19^ by including data for more ion types and for lower doses (5 cGy). The goals of analyzing this enhanced data set included improved model-based quantification of dose–response shapes, NTE and TE model parameters, and radiation quality metrics: traditional relative biological effectiveness (RBE) and, as an alternative, the radiation effects ratio (RER)^19^.

## Methods

### Data sets

We analyzed updated data on intestinal tumorigenesis in male APC^(1638N/+)^ tumor-prone mice exposed at the NASA Space Radiation Laboratory (NSRL) to γ rays, protons, ^4^He, ^12^C, ^16^O, ^28^Si or ^56^Fe ions. This mouse strain is a useful model system because the most common driver mutation for colorectal carcinoma in humans is mutation in the tumor suppressor gene APC ^20^. These data were kindly provided by our collaborators at Georgetown University. The lowest tested dose of heavy ions was 5 cGy, which is relevant for long-duration space missions^1^. The covered LET range of ~ 0.2 to 148 keV/µm is broad and encompasses sparsely ionizing and densely ionizing radiations. The doses, LET values, and numbers of mice for each ion were as follows: unirradiated controls (68 mice), protons (1000 MeV/n; 50 to 120 cGy; 0.22 keV/µm, 40 mice), ^4^He (250 MeV/n; 5 to 50 cGy; 1.6 keV/µm, 92 mice), ^12^C (290 MeV/n; 10 to 200 cGy; 13 keV/µm, 60 mice), ^16^O (325 MeV/n; 5 to 50 cGy; 22 keV/µm, 66 mice), ^28^Si (300 MeV/n; 5 to 140 cGy; 69 keV/µm, 136 mice), ^56^Fe (1000 MeV/n; 5 to 160 cGy; 148 keV/µm, 90 mice), γ rays (5–200 cGy, 127 mice). Details of the experimental methods are described in earlier publications^5,6^.

### Radiation response model

Our radiation carcinogenesis dose response model^19^ combines both the TE and NTE components. The TE component in the APC^(1638N/+)^ mouse system is reasonably described by a linear dependence over the dose range of interest for space missions^19^. In contrast, the NTE component tends to be non-linear with a concave shape, particularly for heavy ions^19^.

In our formalism, the TE and NTE radiation effects are combined in the function *M*, which represents the radiation response for the number of intestinal tumors per mouse. The function is shown in the following equation, where *B* is the background parameter (i.e. tumors in unirradiated mice), D is the radiation dose for the selected radiation type (e.g. Fe ions), *N* and *T* are the respective NTE and TE parameters, and *s* is the NTE “slope” parameter:

1 $$M=B + N \left(1-\mathrm{exp}\left[-s D\right]\right) + T D$$

Here the parameter notation was changed from the previous version described in reference^19^ for easier interpretation. The parameter *T* is the slope of the linear TE dose response component. The *N* parameter is the “plateau” to which the NTE dose response contribution saturates when all susceptible cells in the affected organ respond to NTE signals released by irradiated cells (e.g. those traversed by heavy ion track cores). The *s* parameter can be interpreted as an exponential “slope” or “saturation rate” for the NTE component of the dose response.

### Distribution of tumors per mouse

The recently updated APC^(1638N/+)^ mouse data, which are analyzed here, suggest that the distribution of tumors per mouse is not simple. Both “overdispersion” and “underdispersion”, compared with the Poisson distribution, are encountered in this data set, depending on radiation type and dose. For example, after 0.05 Gy of γ rays, the mean number of tumors/mouse (µ) was 3.84 and variance/mean (V/µ) was 0.57, indicating notable underdispersion relative to the Poisson distribution, where V/µ = 1 by definition. In contrast, after 1.4 Gy of Si ions the mean number of tumors/mouse was 23.28 and V/µ was 4.58, indicating notable overdispersion.

To describe this complexity in the variability of tumor count data, we used the following customized weighted negative binomial (WNB) distribution, where *k* is the number of tumors per mouse, *P*_*WNB*_(*k*) is the probability of *k*, *Γ* is the Gamma function, *M* is the radiation response function (from Eq. 1), *r* and *q* are parameters that describe the variance, and *Y* = *k* + 1/*r*:

2 $${P}_{WNB}\left(k\right)=\frac{{[\left(1+r M\right)}^{-Y} {r}^{k} {M}^{\left(k-1\right)} \Gamma \left(Y\right) \left(M+k q\right)]}{\left[\Gamma \left(1+k\right) \Gamma \left(\frac{1}{r}\right) \left(1+q\right)\right]}$$

The solution for the mean number of tumors per mouse (µ), based on Eq. (2), is as follows:

3 $$\mu =\left[M+q \left(1+M \left(1+r\right)\right)\right]/(1+q)$$

The mean excess tumor yield due to radiation (µ_*rad*_), with background mean tumor yield subtracted, is:

4 $${\mu }_{rad}=[N \left(1-\mathrm{exp}\left[-s D\right]\right)+T D] (1+q \left(1+r\right))/(1+q)$$

The structure of Eq. (4) shows that the radiation-induced tumor yield is proportional to the dose response function *M* (from Eq. 1, without the background parameter *B*), with the proportionality constant depending on the variance parameters *r* and *q*.

The customized WNB error distribution (Eq. 2) allows both underdispersion and overdispersion of tumors per mouse, compared with the Poisson distribution. Variance/mean for the WNB distribution has the following solution:

5 $$\frac{V}{\mu }=\left(1+r M\right) \frac{\left[q+M \left(1+{q}^{2} \left(1+r\right)+2 q \left(1+r\right)\right)\right]}{[\left(1+q\right) \left(q+M \left(1+q \left(1+r\right)\right)\right)]}$$

Taylor series calculations show that at small *M* values, which occur at low radiation doses, V/µ approaches the following underdispersed solution:

6 $$\frac{V}{\mu }=\frac{1}{1+q}$$

In contrast, at large *M* values, which occur at high radiation doses, V/µ becomes progressively more overdispersed (> 1) as *M* increases.

### Model fitting

The main model parameters (*T*, *N*, *s* and *r*) were estimated by fitting the model to the data by maximum likelihood techniques, as described below. Importantly, we sought to reduce the number of freely adjustable parameters to simplify the model structure and the interpretation of model predictions. For this reason, we did not allow the remaining parameters *B* and *q* to be freely adjustable, but instead estimated both of them based on observed data for unirradiated control mice. Based on these assumptions, the following equation describes the relationship of these parameters with the mean background tumor yield (µ_*bac*_, observed value = 3.279).

7 $${\mu }_{bac}=\left[B+q (1+B (1+r))\right]/(1+q)$$

This equation (Eq. 7) can be solved for *B* as function of µ_*bac*_, as follows:

8 $${B}_{sol}=\left[\left({\mu }_{bac}-1\right) q+{\mu }_{bac}\right]/\left[1+q (1+r)\right]$$

The relationship between the parameters of interest and the background variance in tumor counts per mouse (*V*_*bac*_, observed value = 2.625) is described as follows, where *B*_*sol*_ is based on Eq. (8):

9 $${V}_{bac}=\frac{\left[\left(\left(1+{q}^{2} \left(1+r\right)+2 q \left(1+r\right)\right) {B}_{sol}+q\right) \left(1+r {B}_{sol}\right)\right]}{{(1+q)}^{2}}$$

There are two solutions for parameter *q* based on Eq. (9). We discarded one of these solutions, which is always negative. The retained positive solution has a singularity in the positive *r* range, so we approximated it with the following smooth function (*q*_*sol_A*_) of *r*:

10 $${q}_{sol\_A}=500 \left(1-\mathrm{exp}\left[-\frac{\mathrm{exp}\left[1.33+26.85 r-20.95 {r}^{2}+1843.14 {r}^{3}\right]}{500}\right]\right)$$

We substituted *q*_*sol_A*_ from Eq. (10) into Eq. (8) to obtain the following approximate explicit solution for *B* (*B*_*sol_A*_) as function of *r*:

11 $${B}_{sol\_A}=\left[\left({\mu }_{bac}-1\right) {q}_{sol\_A}+{\mu }_{bac}\right]/\left[1+{q}_{sol\_A} (1+r)\right]$$

These solutions for parameters *B* and *q* (*B*_*sol_A*_ and *q*_*sol_A*_ from Eqs. 10–11) were generated for each radiation type, based on the best-fit value of parameter *r*. In other words, the model was substantially simplified by allowing the fitted value for one parameter (*r*) to determine the values of two other parameters (*B* and *q*).

We fitted the model to individual tumor count data for each mouse by maximizing the log likelihood function (*LL*) over all the data for studied radiation types combined (controls, γ rays, H, He, C, O, Si and Fe ions), using the sequential quadratic programming algorithm implemented in Maple 2020 software. To improve biological realism, we implemented the following constraints: (1) All parameters were restricted to non-negative values. (2) The *T* parameter for particle radiations (H, He, C, O, Si and Fe ions) was restricted to be at least as large as the best-fit value for γ rays. This restriction was mainly relevant for those heavy ions (e.g*.* He, O) for which doses > 1 Gy were not available, because such high doses were numerically influential in determining the *T* parameter for those radiations where they were available. To maximize the probability of finding a global (rather than a local) optimum for the model fit, the model was refitted 2000 times using random initial parameter values, and the best-fit solution among all these attempts was selected as the optimum.

To simplify the model further, we assessed whether certain parameters could be kept in common for all radiation types. This was performed by comparing the performances of model variants with a selected parameter kept in common vs. allowed to be different for different radiation types. The comparisons were done using the Akaike information criterion with sample size correction (AICc)^21,22^. We found that parameter *s* could be kept in common for all radiation types, *T* and *r* could be kept in common for γ, H, He, C and O, and different for Si and Fe, whereas parameter *N* differed for each radiation type. This model variant was used for the analyses described below.

### Metrics for comparing the effects of different radiation types: RBE and RER

The traditional metric for comparing the effects of different radiations is the relative biological effectiveness (RBE), defined as the ratio of iso-effective doses for a reference radiation (e.g. γ rays) vs. a radiation of interest (e.g. heavy ions). In the context of radiation carcinogenesis, RBE is the ratio of doses of two different radiations that result in equivalent radiogenic (excess) tumor yields. RBE is easily interpretable, it has a long history of use, and it is convenient to use for simple dose responses such as linear or linear quadratic. However, when radiation effects saturate at high doses or decrease due to cell killing, RBE is sometimes impossible to calculate because not even a large γ ray dose can produce an effect of the same magnitude as a given high-LET radiation dose^19^.

Alternatively, the relative effectiveness of two different radiations can be assessed by comparing the mean radiation-induced tumor yields per exposed animal for one radiation vs. the other, at the same radiation dose. The key difference between this concept, which we called the Radiation Effects Ratio (RER)^19^, and RBE is that the RER compares the effects of the two radiations at the same radiation dose. RER for ion *I*, relative to γ rays, is calculated as follows, where *d* is the radiation dose (the same for ion *I* and γ rays) and µ_*rad*_ is the radiation-induced excess tumor yield (from Eq. 4) for each radiation type at dose *D*:

12 $$RER(I)={\mu }_{rad}(D,I)/{\mu }_{rad}(D,\gamma )$$

In contrast to this simple RER solution (Eq. 12), the RBE solution for our model was more complicated, and involved numerical evaluation of the Lambert W function.

### Uncertainty estimation

Uncertainties (95% confidence intervals, CIs) were estimated for each adjustable model parameter, for model predictions, and for different radiation quality metrics (RBE and RER) using the following Monte Carlo procedure. Multiple (> 7500) randomly-selected parameter value combinations that produced model fits which fell within the 95% CIs of the best fit (assessed by profile likelihood) were generated and stored. Then these parameter combinations were used to generate distributions of each parameter and metric at each dose of interest. The minimum and maximum values of each distribution generated by Monte Carlo were used as estimates for the 95% CIs of the selected parameter. The same procedure was repeated for each dose of interest: 50 dose points evenly spread on a logarithmic scale between 1 mGy and 2.54 Gy were used.

### Incremental effect additivity (IEA) synergy theory calculations

Since astronauts on long-distance space missions will be exposed to a complex mixture of different radiations, including various types and energies of heavy ions, it is important to predict the potential adverse health effects of this radiation mixture, based on the modeled dose responses for each radiation type. It is known, but often overlooked, that the approach of estimating mixture effects by simply adding the effects of each component, called simple effect additivity (SEA), is wrong unless each mixture component’s dose response is approximately linear-no-threshold (LNT)^23^. Applying SEA to dose response shapes that strongly deviate from linearity, which can occur for heavy ion induced carcinogenesis at space-relevant doses^5,19,24,25^, leads to numerically incorrect and misleading predictions for the effect of a mixture of these heavy ions.

To solve this problem, Sachs et al. recently developed an improved synergy theory approach called incremental effect additivity (IEA)^23,26,27^. The IEA methodology uses the dose response of each component radiation to accurately predict the effect of a mixture of these components, under the assumptions of no synergy and no antagonism between components, even for strongly non-linear dose response shapes^23,26,27^. Deviations of measured results from IEA predictions can be interpreted as evidence for synergistic or antagonistic interactions between the effects of different radiation types.

Here we applied IEA to a mixture of H, He, C, O, Si and Fe ions, with proportions and doses for each of these ions relevant for a 940 day-long mission to Mars. The dose estimates for each ion (in Gy) were based on Kim et al.^1^: H = 0.311; He = 0.109; C = 0.029; O = 0.029; Si = 0.022; Fe = 0.019; total = 0.519. The fractional contributions to total dose were, therefore, ~ 60% for H and ~ 21% for He, with the remainder made up of heavier ions. The dose response for each ion was taken from our model (µ_rad_, Eq. 4) with best-fit parameter values. We compared the IEA results with SEA to numerically demonstrate the differences between these approaches using the data and models analyzed here.

The first step of IEA involves calculating the derivative of the dose response function over dose. The solution is:

13 $$\frac{{d\mu }_{rad}}{dD}=(N\ s\ \mathrm{exp}[-s D] + T) (q r + q + 1)/(1 + q)$$

The next IEA step involves calculating the inverse function for the dose response. The inverse function represents the radiation dose needed to produce a mean biological effect (µ_rad_) of a specified magnitude (labeled *v* here). The solution for the inverse function is described by the following equation, where *LW* is the Lambert W function, *Q*_1_ = *T* [1 + *q* (*r* + 1)] and *Q*_2_ = [(-(*r* + 1) *N* + *v*) *q* + *v* – *N*]:

14 $${F}_{inv}=({Q}_{1} LW[\mathrm{exp}\left[-s \frac{{Q}_{2}}{{Q}_{1}}\right] N \frac{s}{T}] + {Q}_{2} s)/({Q}_{1} s)$$

Using the inverse function (Eq. 14) with best-fit parameter values relevant to each studied radiation type, we then calculated what dose magnitude is needed for each radiation type to produce the total effect observed for the radiation mixture. These solutions were then substituted into dose response function derivatives (Eq. 13) for each radiation type. All these component calculations were then combined into a differential equation, which was solved numerically to generate effect predictions for the Mars mission-relevant radiation mixture.

We calculated the uncertainties of IEA results for the selected Mars mission-relevant radiation mixture dose, considering uncertainties and correlations of the model parameters for individual ion dose responses. This task was performed by applying the IAE methodology to each of the stored model parameter combinations that fell within the 95% CI region of the original model fit described above.

## Results

**Figure 1:**
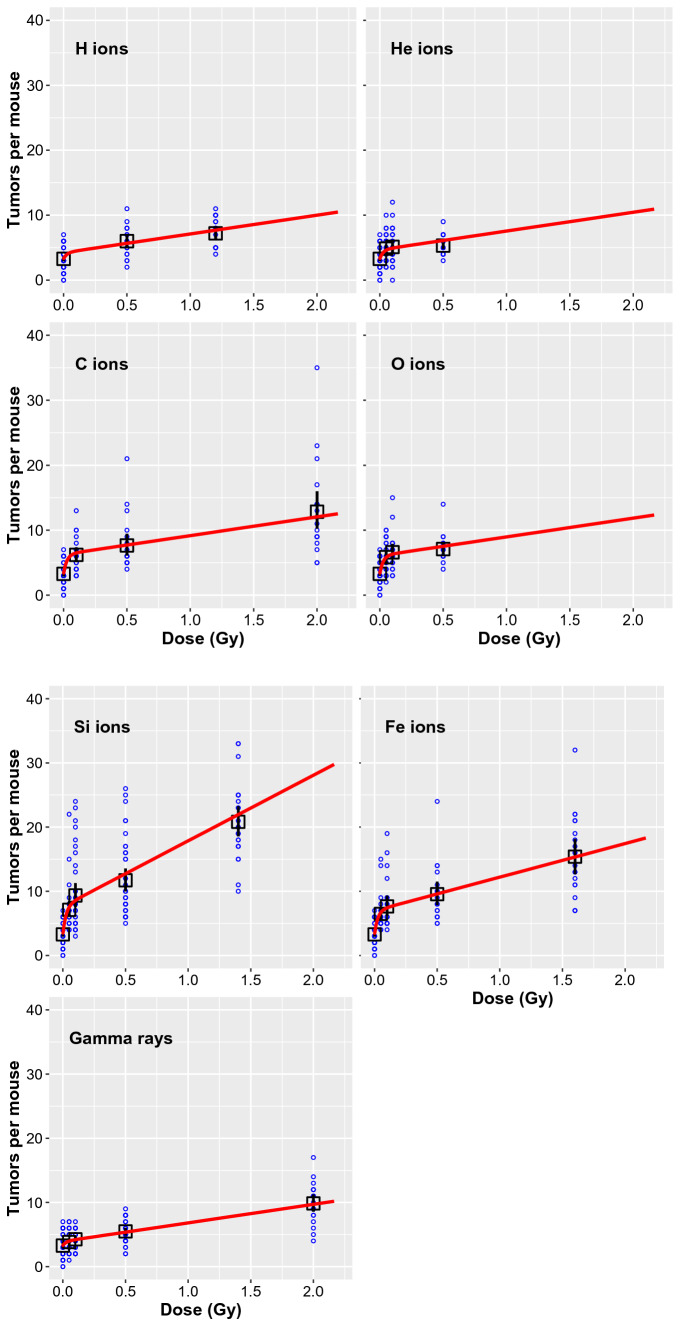
Comparison of model fits (red curves) with the data (blue circles) for mouse tumorigenesis induced by H, He, C, O, Si, or Fe ions, or γ rays. Blue circles represent the number of tumors in individual mice. Black squares and bars represent mean values and standard errors, and red curves represent model fits. Two of the Si irradiated mice had > 40 tumors (50 and 53), but these points are not shown in the Si panel below to improve visualization at lower y-axis values.

The best-fit modeled radiation responses for mouse tumorigenesis induced by H, He, C, O, Si and Fe ions are shown in Fig. 1. Comparison of these dose responses with the data show that the main non-linearity occurs at low doses, in the range of 0, 0.05 and 0.1 Gy. The non-linearity is particularly prominent for the heavier ions (Si and Fe), and is attributed to NTE by our model.

**Figure 2:**
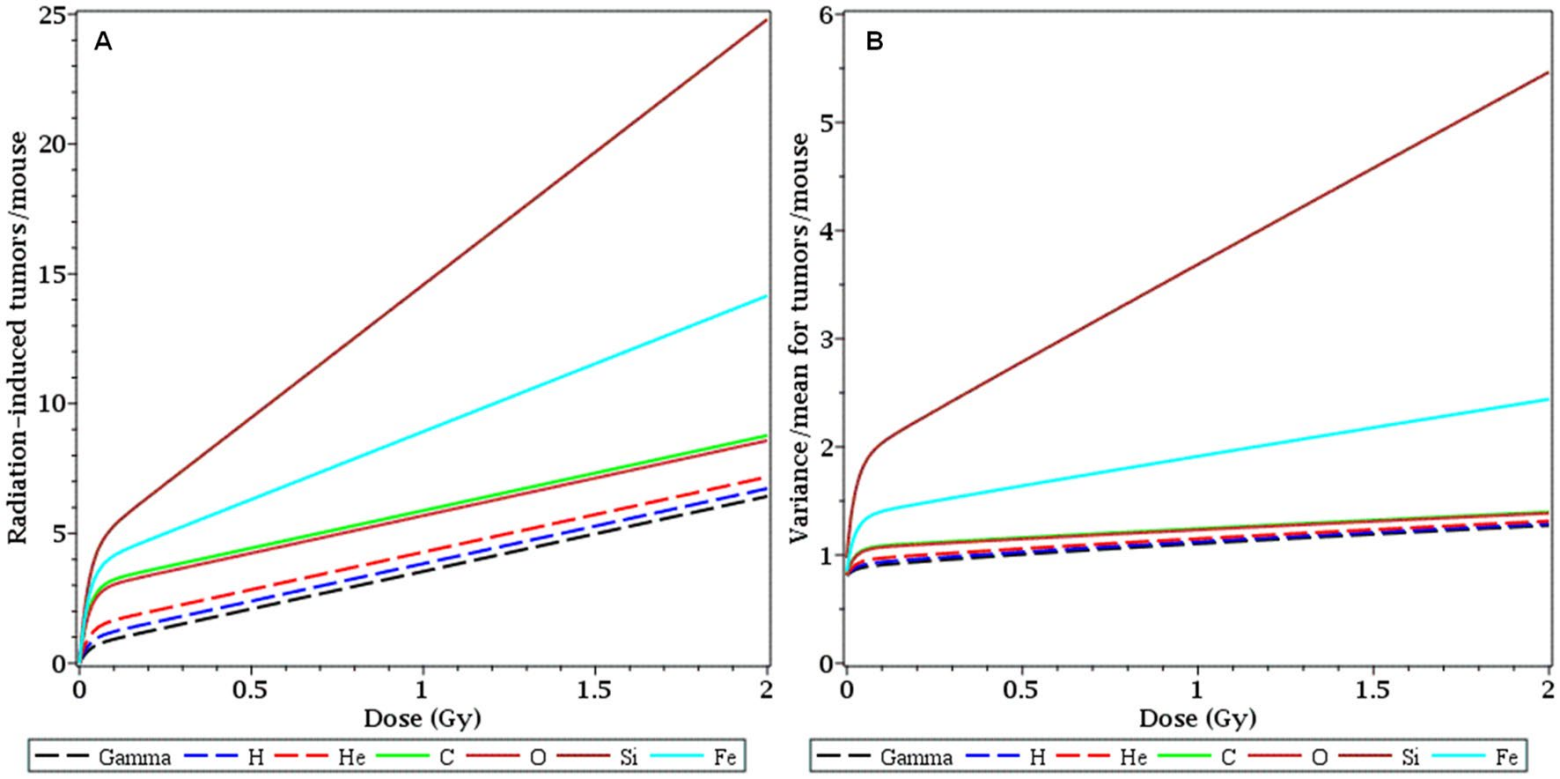
Comparison of best-fit radiation dose responses for the mean number of radiation-induced tumors per mouse (panel A) and for the variance/mean (panel B) for each radiation type.

The best-fit dose responses for all radiation types are compared next to each other on the same plot in Fig. 2. The mean number of radiation-induced tumors per mouse is shown in Fig. 2A, and variance/mean is shown in Fig. 2B. These curves clearly indicate that Si and Fe ions were the most effective at tumorigenesis, among the studied radiation types (Fig. 2A). These two ion types were also associated with the largest degree of overdispersion of tumor counts per mouse, compared with the Poisson distribution (i.e. variance/mean > 1, Fig. 2B). These results suggest that Si and Fe ions produce the steepest and most non-linear dose responses with the greatest degree of overdispersion (i.e. some mice exposed to these ions had very high numbers of tumors, much larger than the mean).

Clarifications of the dose response shapes and structure in the space-relevant dose region were made possible in the current study due to the availability of new data at 5 cGy for most of the tested radiation types, whereas our earlier analysis^19^ was based on doses ≥ 10 cGy. The findings of nonlinear dose response shapes at low doses strengthen the interpretation that NTE can play an important role in detrimental effects of space radiations.

**Figure 3:**
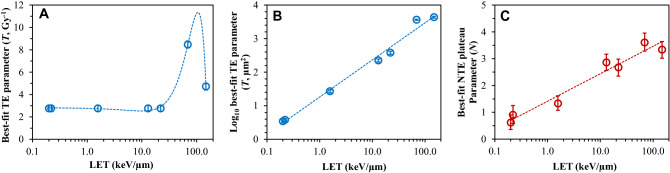
Dependences of the TE and NTE model parameters on radiation LET. Symbols represent best-fit parameter values for each radiation type. Lines are polynomial fits shown for visualization only. **(A)** TE parameter (*T*) expressed per unit dose. **(B)** TE parameter expressed per radiation fluence using the dose/fluence conversion coefficient. **(C)** NTE parameter (*N*) expressed per unit dose. Error bars represent 95% CIs.

The best-fit model parameter values and their uncertainties are shown in Table 1 and Fig. 3. The NTE slope parameter (*s*) was kept in common for all radiation types, which simplified the formalism compared with our earlier analysis^19^. The best-fit *T* and *N* parameter values tended to increase with increasing radiation LET (Fig. 3). The *T* parameter expressed as function of dose (Gy^-1^) tended to be relatively stable over the LET range of < 22 keV/µm, then increased, but subsequently peaked at ~ 69 keV/µm (Si ions) and decreased again at even higher LET values (Fig. 3A). We also expressed the *T* parameter as function of ion fluence per µm^2^ based on the simple fluence/dose relationship using the eV to J conversion coefficient (Table 1, Fig. 3B). This conversion showed that the *T* parameter increased steadily with increasing LET with potential saturation at very high LETs, probably due to sparsity of ion core “hits” per cell for very heavy ions at relevant doses. In comparison, the *N* parameter exhibited a smoother continuous increase, roughly as function of the logarithm of LET (Fig. 3C). Notably, the *N* parameter was not consistent with zero even for γ rays and protons in this analysis, which is in agreement with reports of NTE even for low-LET radiations^28,29^. The overdispersion parameter *r* was maximal for Si ions (Table 1), similarly to what was found in our previous analysis^19^.

**Table 1:** Best-fit model parameter values and uncertainties for each studied radiation type.

Radiation type	LET (keV/µm)	TE parameter (*T*, Gy^−1^)			NTE parameter (*N*)			Variance parameter (*r*)		
			95% CI			95% CI			95% CI	
γ		2.77	2.47	3.04	0.62	0.33	0.87	0.047	0.021	0.077
H	0.22	2.77	2.47	3.04	0.89	0.58	1.18	0.047	0.021	0.077
He	1.57	2.77	2.47	3.04	1.34	1.08	1.62	0.047	0.021	0.077
C	13	2.77	2.47	3.04	2.87	2.56	3.20	0.047	0.021	0.077
O	22	2.77	2.47	3.04	2.68	2.34	2.94	0.047	0.021	0.077
Si	69	8.52	8.20	8.85	3.58	3.28	3.90	0.208	0.143	0.301
Fe	148	4.74	4.42	5.07	3.34	3.05	3.63	0.107	0.054	0.193

As described in the main text, parameter *s* was assumed to be the same for all radiation types, and its best-fit value was 38.66 (95% CI: 38.34, 38.98) Gy^−1^. The detailed assumptions about other parameters, some of which were kept in common for several radiation types, are also described in the main text.

**Figure 4:**
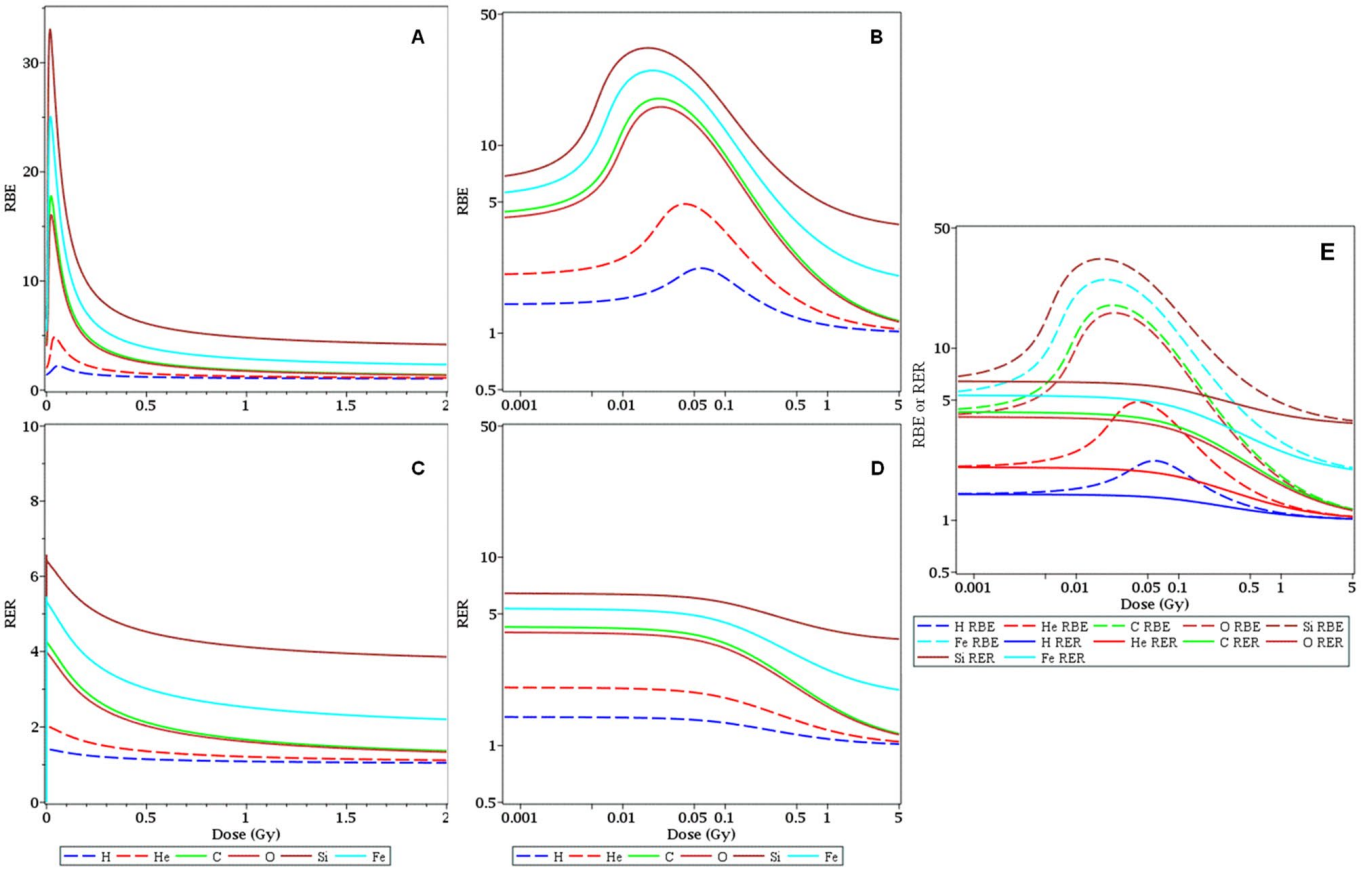
Relative biological effectiveness (RBE, **A,B**) and radiation effects ratio (RER, **C,D**) values for each ion type, relative to γ rays. These metrics were calculated based on best-fit model parameter values for each radiation type. They are compared to each other in **(E)**.

**Figure 5:**
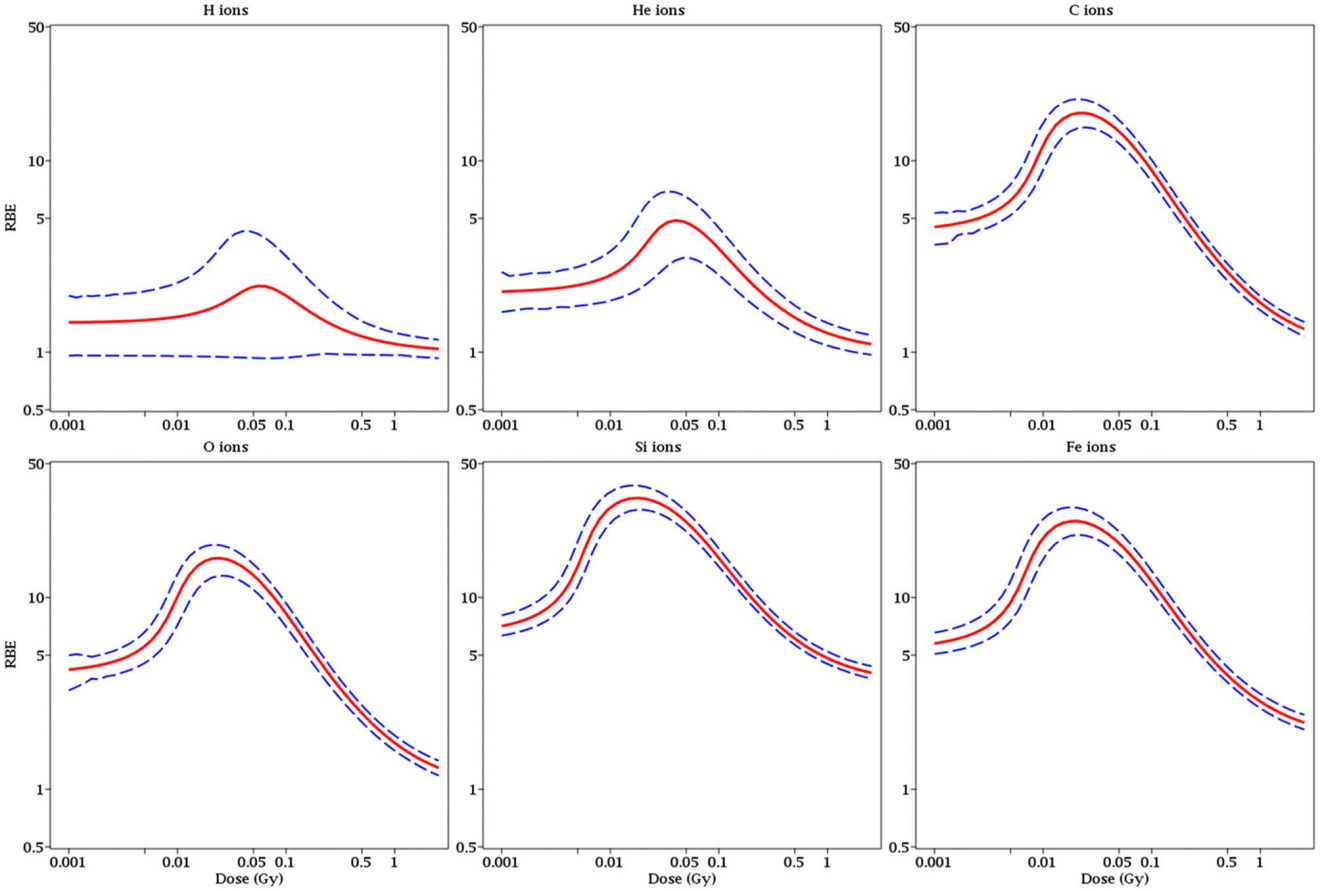
Visualization of uncertainties (95% CI, blue dashed curves) around model-predicted RBE values (red curves) for each ion type.

**Figure 6:**
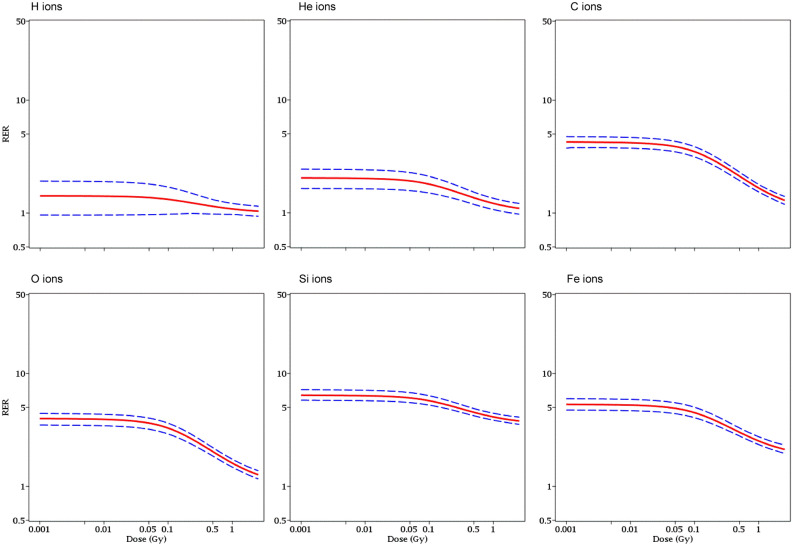
Visualization of uncertainties (95% CI, blue dashed curves) around model-predicted RER values (red curves) for each ion type.

Estimates of the dose-dependent RBE and RER metrics for each radiation type, along with their uncertainties (95% CI), are shown in Figs. 4, 5 and 6. In the context of our model, RBE and RER are asymptotically the same at very low doses where NTE terms dominate, and also at very high doses where TE terms dominate. However, these metrics differ at intermediate doses, where RBE is greater than RER (Fig. 4E). Notably, the RBE and RER estimates were modified compared with our earlier analysis^19^ due to enhanced data set size and level of detail. Their 95% CI bands were also narrowed (Figs. 5 and 6) because of the clarification provided by the new data at 5 cGy doses and by the improved modeling of the data variability by the customized WNB error distribution.

Since astronauts who travel on interplanetary space missions such as a trip to Mars and back will be exposed to a mixture of different radiation types, it is important to use the information on dose response shapes and magnitudes for individual components of this mixture to predict the effects of the entire mixture. A novel and powerful method for generating such predictions, under the assumptions of no synergy and no antagonism between the effects of all components, was developed by Sachs et al.^23,26,27^. This new approach, called incremental effect additivity (IEA), is reliable for non-linear dose response shapes, such as those encountered in this analysis. In contrast, the often used simple effect additivity (SEA) approach is incorrect for non-linear dose response shapes^23,26,27^. Here we compared both methods for a Mars mission-relevant mixture of ions (based on reference^1^), as described in the Materials and Methods section.

**Figure 7:**
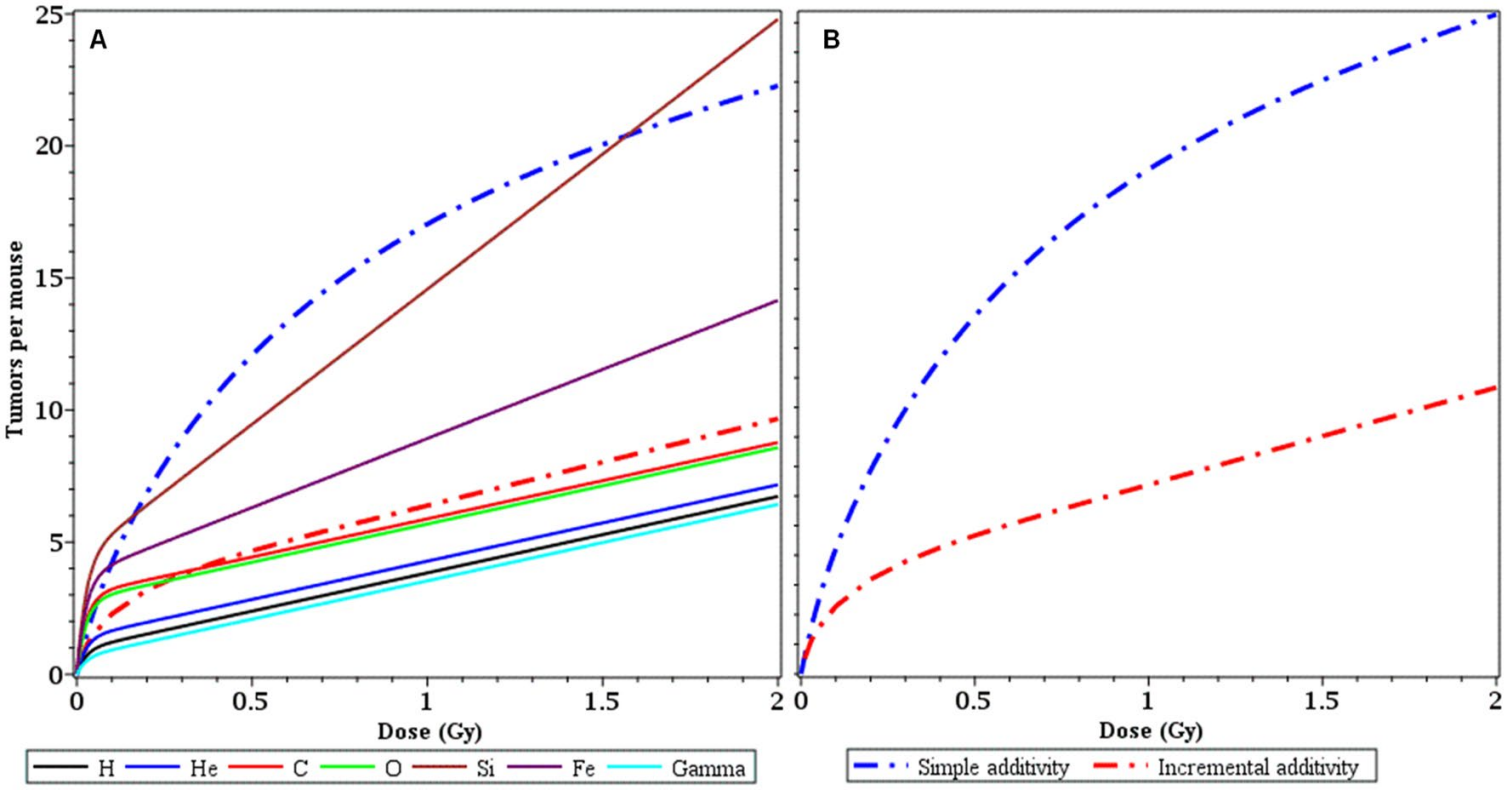
Comparison of predictions for mean radiation-induced tumors per mouse by the simple effect additivity (SEA, blue dashed curves) and incremental effect additivity (IEA, red dashed curves) for a mixture of ion types with proportions relevant for a mission to Mars. **(A)** IEA predictions fall between the dose responses of individual ions, as intuitively expected. In contrast, SEA predictions incorrectly overestimate the tumor yield. **(B)** IEA and SEA predictions next to each other. The details of both methods are described in the main text.

The results of these IEA and SEA analyses are compared in Fig. 7. They clearly show that SEA results are incorrect and greatly overestimate the dose response for the mixture of ions, such that the predicted SEA mixture response wrongly exceeds even the dose response for the most biologically effective Si ion component (Fig. 7A). In comparison, IEA results for mixture effects plausibly fall within the range of individual ion dose responses. At an estimated total dose for the ion mixture on a Mars mission (0.519 Gy) IAE predicts 4.74 excess tumors per mouse and SEA predicts 12.33 tumors, which is 2.6-fold higher.

**Figure 8:**
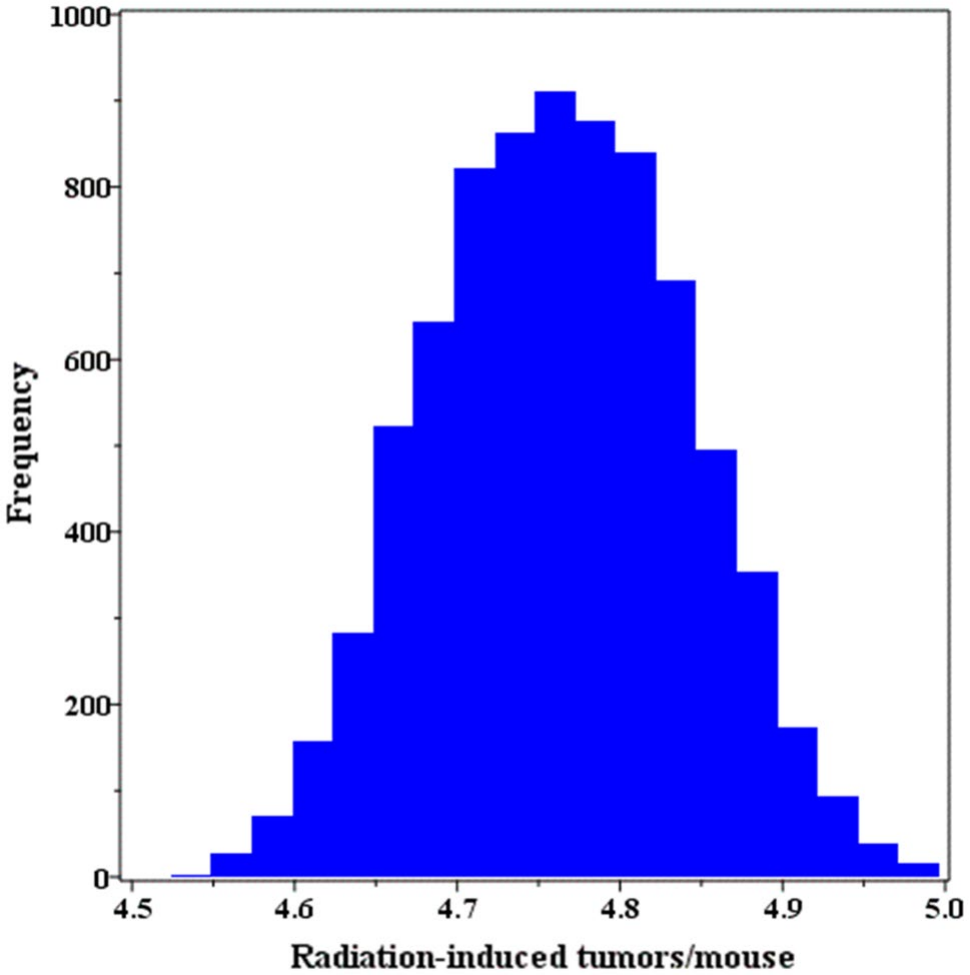
A histogram of incremental effect additivity (IEA) predictions for radiation-induced tumors per mouse for a mixture of ion types with proportions and doses relevant for a mission to Mars. This histogram is composed on Monte Carlo repeats of the IEA procedure on > 7500 model parameter combinations that fell within the 95% confidence region of the model fit, based on profile likelihood. Details are described in the main text.

Uncertainties for the IEA predictions were estimated by repeating the procedure for multiple (> 7500) model parameter combinations that fell within the 95% confidence region of the model fit. These uncertainties are shown as a histogram in Fig. 8, and ranged from 4.50 to 5.00 excess tumors per mouse. These results suggest that, despite considerable uncertainties associated with best-fit model parameters, the resulting IEA prediction uncertainties are not excessively large.

## Discussion

The current analysis substantially extends our previous published results^19^ by using a larger updated data set, which now includes lower dose (5 cGy) data for most tested radiation types. A more detailed modified model formalism that uses the WNB distribution to better describe the data variability was developed and implemented on this data set. This new information and improved modeling approach allowed better estimation of dose response shapes in the space-relevant low dose region. The best-fit dose response for each radiation type was a smooth function, asymptotically linear at very low doses where NTE dominate and also asymptotically linear at high doses where TE begin to dominate (Figs. 1 and 2). Importantly, these dose response shapes were non-linear (concave) at intermediate doses, which are relevant for space travel (Figs. 1 and 2). The non-linearity was interpreted by our model as a combination of TE and NTE components, each of which can have a different dependence on radiation type (Fig. 3).

Due to updates to the data set and modeling approach, RBE and RER values for heavy ions and protons were estimated with more information than previously possible (Figs. 4, 5 and 6). RBE and RER were asymptotically the same at very low and very high doses, but RBE exceeded RER by up to several-fold in the intermediate space-relevant dose range of 1–50 cGy (Fig. 4E).

We used the modeled dose responses for each radiation type to predict the biological effect of a mixture of these radiations. This was done using the new and powerful IEA synergy theory^23,26,27^ for a mixture with radiation type proportions relevant for a mission to Mars. The results (Figs. 7 and 8) demonstrate that the IEA approach can be combined with our modeling methodology (Eq. 1) to generate quantitative predictions for space radiation mixture effects, under the assumptions of no synergy and no antagonism. These predictions can subsequently be tested experimentally.

In conclusion, we believe that the proposed modeling approach can enhance current knowledge about quantification of health risks from space radiation. The modeled dose responses for individual radiation types can also serve as a basis for predicting the risks from complex multi-component radiation mixtures to examine potential deviations from additivity in the biological effects of such mixtures.
